# Using ecological niche modeling to predict the potential distribution of scrub typhus in Fujian Province, China

**DOI:** 10.1186/s13071-023-05668-6

**Published:** 2023-01-31

**Authors:** Xuan Li, Xianyu Wei, Wenwu Yin, Ricardo J. Soares Magalhaes, Yuanyong Xu, Liang Wen, Hong Peng, Quan Qian, Hailong Sun, Wenyi Zhang

**Affiliations:** 1 Department of Epidemiology and Biostatistics, School of Public Health, Anhui Medical University, Hefei, China; 2 Chinese PLA Center for Disease Control and Prevention, Beijing, China; 3 Chinese Center for Disease Control and Prevention, Beijing, China; 4 Spatial Epidemiology Laboratory, School of Veterinary Science, The University of Queensland, Brisbane, Australia; 5 Child Health Research Center, The University of Queensland, Brisbane, Australia

**Keywords:** Scrub typhus, Risk factors, Negative binomial regression, Maximum entropy modeling

## Abstract

**Background:**

Despite the increasing number of cases of scrub typhus and its expanding geographical distribution in China, its potential distribution in Fujian Province, which is endemic for the disease, has yet to be investigated.

**Methods:**

A negative binomial regression model for panel data mainly comprising meteorological, socioeconomic and land cover variables was used to determine the risk factors for the occurrence of scrub typhus. Maximum entropy modeling was used to identify the key predictive variables of scrub typhus and their ranges, map the suitability of different environments for the disease, and estimate the proportion of the population at different levels of infection risk.

**Results:**

The final multivariate negative binomial regression model for panel data showed that the annual mean normalized difference vegetation index had the strongest correlation with the number of scrub typhus cases. With each 0.1% rise in shrubland and 1% rise in barren land there was a 75.0% and 37.0% increase in monthly scrub typhus cases, respectively. In contrast, each unit rise in mean wind speed in the previous 2 months and each 1% increase in water bodies corresponded to a decrease of 40.0% and 4.0% in monthly scrub typhus cases, respectively. The predictions of the maximum entropy model were robust, and the average area under the curve value was as high as 0.864. The best predictive variables for scrub typhus occurrence were population density, annual mean normalized difference vegetation index, and land cover types. The projected potentially most suitable areas for scrub typhus were widely distributed across the eastern coastal area of Fujian Province, with highly suitable and moderately suitable areas accounting for 16.14% and 9.42%, respectively. Of the total human population of the province, 81.63% reside in highly suitable areas for scrub typhus.

**Conclusions:**

These findings could help deepen our understanding of the risk factors of scrub typhus, and provide information for public health authorities in Fujian Province to develop more effective surveillance and control strategies in identified high risk areas in Fujian Province.

**Supplementary Information:**

The online version contains supplementary material, which is available at 10.1186/s13071-023-05668-6.

## Background

Scrub typhus, a vector-borne disease caused by *Orientia tsutsugamushi*, is prevalent in the Tsutsugamushi Triangle [1]. The transmission mode of scrub typhus to humans is generally through the bite of infected chiggers of the genus *Leptotrombidium* [2]. Rodents are the main reservoir, and their mites act as the vector of the disease [3]. Following infection through the bite of an infected chigger, typical clinical manifestations include an eschar, fever, headache, rash and lymphadenopathy [4, 5]. In certain patients infection may lead to multiple organ failure or even death [6]. Over the past decades, scrub typhus has become a significant public health problem in China due to the expansion of areas endemic for the disease and increases in its incidence and infection risk [7].

Fujian Province is historically an endemic area for scrub typhus in China [8]. In our previous study [9], which explored the demographic characteristics and spatiotemporal dynamics of scrub typhus cases from January 2012 to December 2020 in Fujian Province, both the frequency and geographic distribution of cases increased dramatically. However, one limitation in that study is that risk factors related to scrub typhus are not contained. Meteorological, socioeconomic, and land cover variables have been shown to be risk factors for scrub typhus, but few studies have analyzed the effects of all three in Fujian Province simultaneously. Therefore, the objectives of this study were to identify the impact of these variables on the occurrence of scrub typhus in Fujian Province, map the areas potentially at risk of its occurrence, and estimate the size of the population at risk, to provide insights into the public health burden of scrub typhus in the province.

## Methods

### Study areas

**Fig. 1:**
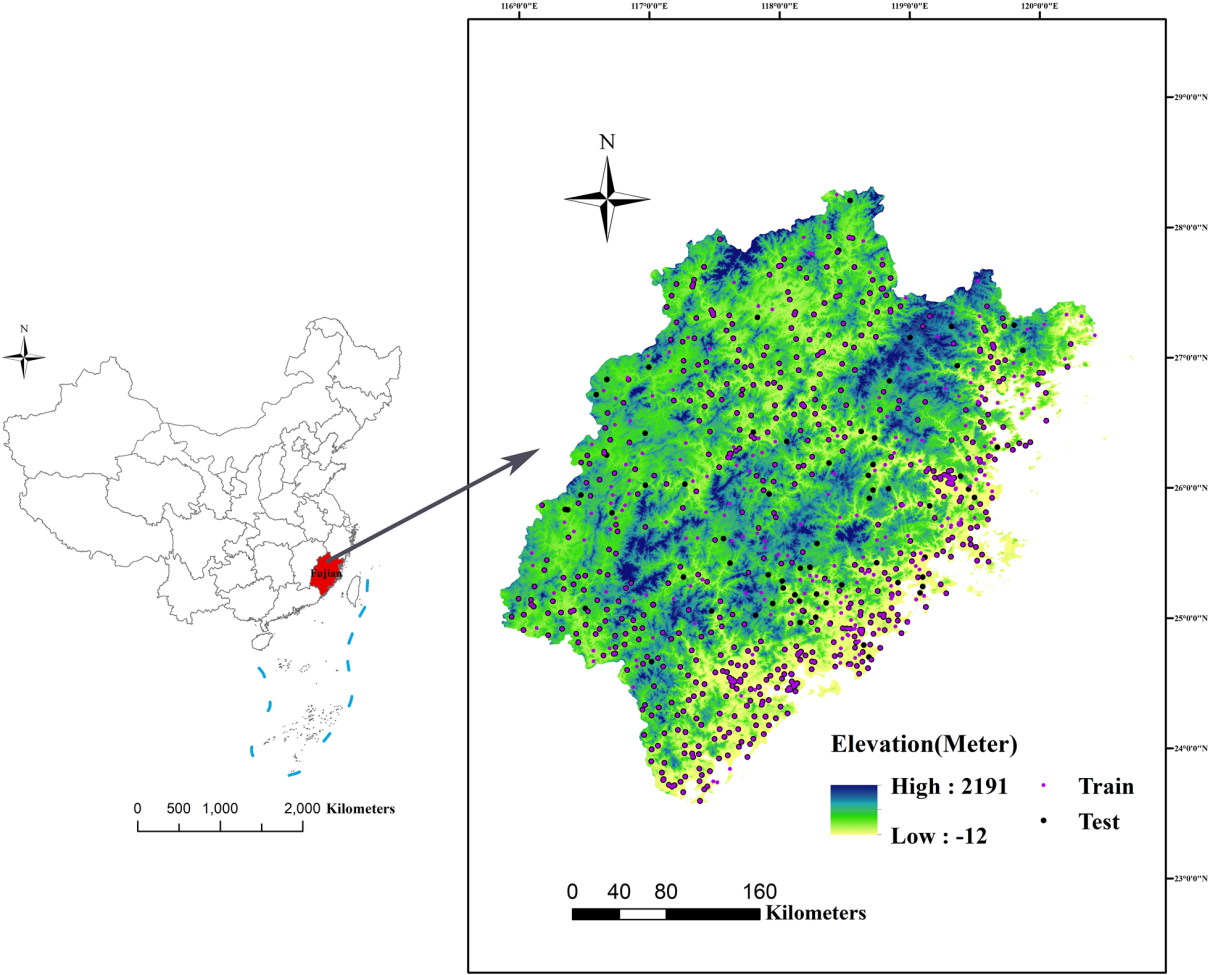
Study area showing the locations of reported cases of scrub typhus, and elevation gradient, of Fujian Province, China

Fujian Province (23°33′–28°20′N, 115°50–120°40′E) is located on the southeastern coast of China and has a total land area of 12.4 million km^2^ (Fig. 1). The climate is characterized as subtropical monsoon, with an average annual temperature of 17–21 °C and average annual rainfall of 1400–2000 mm. The average annual wind speed from south to north along the coast ranges from 6.56 to 9.07 m/s. The human population comprised 4161 million residents at the end of 2020 according to the 2021 Fujian statistical yearbook. The coastal areas have a relatively high population density. Mountainous and hilly land accounts for 80% of the total area of the province, and is mainly located in central and western areas. Fujian Province comprises eight main land cover types, of which forest and cropland account for 95.30% of the total area; the remaining types are: impervious surfaces, water bodies, grassland, shrubland, barren land and wetlands.

### Data collection and management

We extracted scrub typhus cases for the period 2012–2020 from the Chinese National Notifiable Infectious Disease Reporting Information System. Monthly scrub typhus cases were used for the analysis. All of the cases of scrub typhus had been diagnosed according to the unified diagnostic criteria of the Chinese Center for Disease Control and Prevention [9, 10].

The annual demographic data at the county level were acquired from the provincial Bureau of Statistics in Fujian (http://tjj.fujian.gov.cn/). A county-level map of Fujian Province at 1:1,000,000 scale was obtained from the Data Center for Geographic Sciences and Natural Resources Research, Chinese Academy of Sciences (http://www.resdc.cn/). The data sources for the environmental variables, including meteorological and land cover variables, and the socioeconomic variables, are shown in Table 1. Monthly meteorological data, county-level socioeconomic data and land cover data were used. There can be a delay in the effect of meteorological variables on scrub typhus due to its 10- to 12-day incubation period and the 2- to 3-month life cycle of chiggers [11]. Therefore, to examine the delay in the effects of meteorological variables, we included a time lag of 0–3 months. Kriging interpolation and Zonal Statistics in ArcGIS software (version 10.6; ESRI, Redlands) were used to calculate average values of each meteorological variable for each county.

**Table 1:** Data sources for the variables used in the models of scrub typhus

Variable category	Variable abbreviation	Variable	Sources
Climate	EVP	Mean evaporation (mm)	China Meteorological Data Service Centre (www.data.cma.cn)
	GST	Mean ground surface temperature (℃)	China Meteorological Data Service Centre (www.data.cma.cn)
	PRS	Mean atmospheric pressure (hPa)	China Meteorological Data Service Centre (www.data.cma.cn)
	RHU	Mean relative humidity (%)	China Meteorological Data Service Centre (www.data.cma.cn)
	SSD	Mean sunshine duration (h)	China Meteorological Data Service Centre (www.data.cma.cn)
	TEM	Mean air temperature (℃)	China Meteorological Data Service Centre (www.data.cma.cn)
	WIN	Mean wind speed (m/s)	China Meteorological Data Service Centre (www.data.cma.cn)
	PRE	Cumulative precipitation (mm)	China Meteorological Data Service Centre (www.data.cma.cn)
Socioeconomic	Dep	Dependency ratios (1%) (young, 0–14 years; old, 65 +)	Open Spatial Demographic Data and Research (www.worldpop)
	Dep_Old	Dependency ratios of old age (1%) (old, 65+ years)	Open Spatial Demographic Data and Research (www.worldpop)
	Dep_Yong	Dependency ratios of young age (1%) (young, 0–14 years)	Open Spatial Demographic Data and Research (www.worldpop)
	Pop_den	Population density [Pop_den (10^3^ persons/km^2^)]	Open Spatial Demographic Data and Research (www.worldpop)
	GDP	Gross domestic product (10^4^ RMB/person)	Resource and Environment Science and Data Center (www.resdc.cn)
Topographical/land cover	Slope	Slope (°)	Geospatial data cloud (www.gscloud.cn)
	Dem	Digital elevation model, elevation (100 m)	Geospatial data cloud (www.gscloud.cn)
	NDVI	Annual mean normalized difference vegetation index	Resource and Environment Science and Data Center (www.resdc.cn)

*RMB* Chinese renminbi

### Correlation between risk factors and scrub typhus cases

Meteorological, socioeconomic and topographical factors and monthly scrub typhus cases were included in the analyses. A negative binomial regression analysis for panel data was performed to explore the relationship between different variables and scrub typhus. In a negative binomial regression, the variance is greater than the mean, which indicates over dispersion. Our data were consistent with this, and were therefore subjected to factor analysis (STATA 17.0 software; StataCorp, College Station, TX) [12].

First, we applied Pearson correlation to analyze the association between different variables; however, highly correlated variables (Pearson correlation coefficients > 0.75) cannot be incorporated into the model at the same time [13]. As the variables showed collinearity and the meteorological variables had different delayed effects with lags of 0–3 months, we selected the variables from the univariate model with the lowest Akaike information criterion for entry into the multivariate model. A *P*-value of < 0.001 was considered to indicate statistical significance. The incidence rate ratio (IRR) was used to reflect the percentage of scrub typhus cases that increased or decreased with change in each variable.

### Maximum entropy modeling procedure

We used the maximum entropy (MaxEnt) model, a machine learning method with a high degree of predictive power [14]. The model combines presence data with background data points of environmental variables to generate habitat suitability predictions [15, 16]. It is based on the theory of maximum entropy, where the largest entropy distribution is considered the optimal distribution and most likely closest to its true state [17]. We employed ecological niche modeling in MaxEnt software 3.4.3 (https://biodiversityinformatics.amnh.org/open_source/maxent/) to explore potential risk factors for the presence of scrub typhus and to predict risk of the disease. Highly correlated variables from the negative binomial regression analysis were screened out from inclusion in the MaxEnt model. Cases of scrub typhus were divided into two partitions: 70% of cases were selected to construct the model (purple dots, Fig. 1), and the remaining 30% were used to validate the model (black dots, Fig. 1). Ten replicate runs of each training partition were used to prevent random errors, and the results averaged [18, 19]. The other modules were set as defaults. The logistic output of the MaxEnt model varies from 0 to 1, and indicates the suitability of an environment for the disease; higher values indicate that a condition is more conducive to the presence of disease [16].

The response curves indicated the relationship between different variables and the predicted probability of presence of the disease. A threshold-dependent binomial test was performed to test the statistical significance of the model using the extrinsic omission rate as a statistic to provide information on model variance and overfitting [20, 21]. To evaluate the validity of the model, we calculated the area under the curve (AUC) of the receiver operating characteristic curve; highest values (closest to 1) are usually considered to indicate the best performing models [22]. The value of AUC ranges from 0 to 1, where AUC = 0.5 is considered to be completely random, values of 0.5–0.6 are considered poor, 0.7–0.8 fair, 0.8–0.9 good, and 0.9–1 excellent [23, 24]. The jackknife test indicates the amount of gain obtained from the combination of all variables or isolated variables [25]. Variables with the highest gain were generally considered to provide the most useful information. Every variable had two gains, of different length and color. The environmental suitability maps had values ranging from 0 to 1, where values < 0.299 were reclassified as unsuitable, 0.29–0.45 as moderately suitable, and > 0.45 as highly suitable [26]. Finally, we overlaid the final suitability map from the reclassified values with human population raster data to estimate areas and populations exposed to different levels of infection risk at the overall level and at the county level [27].

## Results

### Correlation between risk factors and scrub typhus cases

**Fig. 2:**
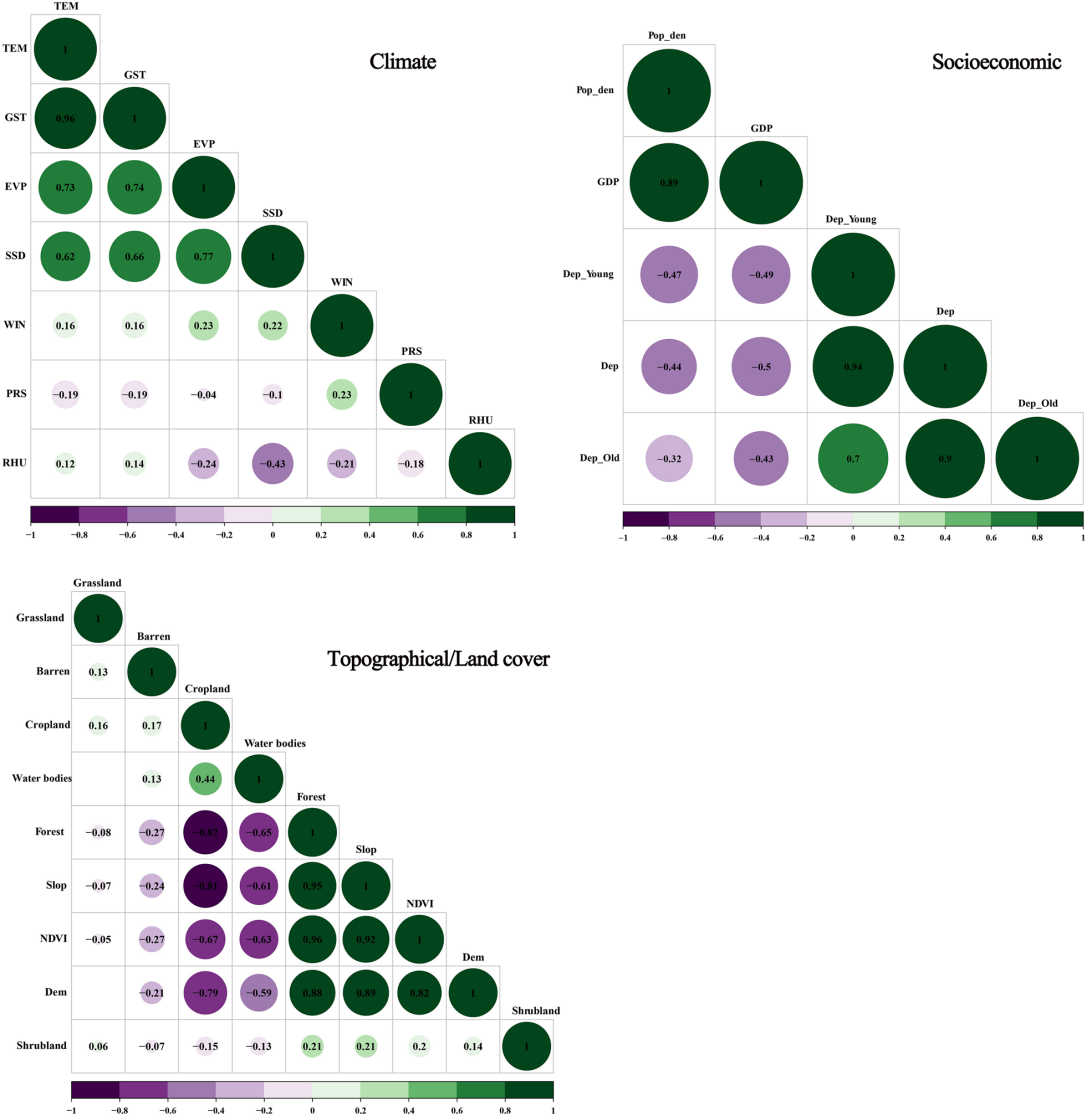
Heat map for Fujian Province indicating Pearson’s correlation coefficients between different environmental variables

Pearson correlations were used to examine the relationships between different environmental variables (Fig. 2). Among the meteorological variables, mean air temperature (TEM) was significantly associated with mean ground surface temperature (GST), and mean evaporation (EVP) was highly correlated with mean sunshine duration (SSD); based on the Akaike information criteria, our model included TEM and EVP. Similarly, Slope, Digital Elevation Model (Dem), Cropland and Forest were excluded from the land cover variables. However, the dependency ratio of old age (Dep_Old) was included in the socioeconomic factors, based on the results of our previous study [9]. The data used in the screening process are shown in Table 2. As a result of the screening method, 12 factors in all were entered into the multivariable regression model.

**Table 2:** Relationships between monthly cases of scrub typhus and potential influencing factors shown by univariate negative binomial regression for panel data

Variables (unit)	Coefficient (95% CI)	IRR (95% CI)	*P*-value	AIC
Climate				
EVP_lag0^a^ (mm)	0.52 (0.48, 0.56)	1.67 (1.61, 1.74)	< 0.00	24,375.89
EVP_lag1 (mm)	0.45 (0.40, 0.49)	1.56 (1.50, 1.63)	< 0.00	24,588.20
EVP_lag2 (mm)	0.31 (0.27, 0.35)	1.36 (1.30, 1.41)	< 0.00	24,828.49
EVP_lag3 (mm)	0.14 (0.10, 0.18)	1.15 (1.11, 1.20)	< 0.00	25,012.46
GST_lag0 (℃)	0.11 (0.10, 0.12)	1.12 (1.11, 1.12)	< 0.00	23,931.92
GST_lag1 (℃)	0.13 (0.12, 0.14)	1.14 (1.13, 1.15)	< 0.00	23,729.46
GST_lag2 (℃)	0.11 (0.10, 0.12)	1.12 (1.11, 1.12)	< 0.00	24,196.09
GST_lag3 (℃)	0.05 (0.04, 0.06)	1.05 (1.04, 1.06)	< 0.00	24,876.57
PRS_lag0 (kPa)	0.09 (0.06, 0.13)	1.10 (1.06, 1.34)	< 0.00	25,036.94
PRS_lag1 (kPa)	0.07 (0.04, 0.10)	1.07 (1.04, 1.11)	< 0.00	25,052.50
PRS_lag2 (kPa)	0.11 (0.07, 0.14)	1.11 (1.08, 1.45)	< 0.00	25,029.30
PRS_lag3^a^ (kPa)	0.17 (0.14, 0.20)	1.19 (1.15, 1.22)	< 0.00	24,949.94
RHU_lag0 (10%)	0.10 (0.02, 0.18)	1.11 (1.02, 1.20)	0.01	25,063.86
RHU_lag1 (10%)	0.47 (0.39, 0.56)	1.60 (1.47, 1.74)	< 0.00	24,950.67
RHU_lag2^a^ (10%)	0.49 (0.40, 0.58)	1.64 (1.50, 1.79)	< 0.00	24,951.69
RHU_lag3 (10%)	0.44 (0.35, 0.53)	1.56 (1.43, 1.71)	< 0.00	24,976.68
SSD_lag0 (h)	0.30 (0.27, 0.32)	1.34 (1.31, 1.38)	< 0.00	24,563.29
SSD_lag1 (h)	0.20 (0.17, 0.23)	1.22 (1.19, 1.26)	< 0.00	24,863.37
SSD_lag2 (h)	0.12 (0.09, 0.14)	1.12 (1.09, 1.15)	< 0.00	24,998.14
SSD_lag3 (h)	0.01 (− 0.01, 0.04)	1.01 (0.99, 1.04)	0.34	25,069.25
TEM_lag0 (℃)	0.14 (0.13, 0.14)	1.15 (1.14, 1.15)	< 0.00	23,834.04
TEM_lag1^a^ (℃)	0.16 (0.16, 0.17)	1.18 (1.17, 1.19)	< 0.00	23,512.24
TEM_lag2 (℃)	0.13 (0.13, 0.14)	1.14 (1.13, 1.15)	< 0.00	24,121.15
TEM_lag3 (℃)	0.06 (0.05, 0.06)	1.06 (1.05, 1.07)	< 0.00	24,888.07
WIN_lag0 (m/s)	− 0.34 (− 0.44, − 0.24)	0.71 (0.64, 0.79)	< 0.00	25,029.24
WIN_lag1 (m/s)	− 0.58 (− 0.69, − 0.48)	0.56 (0.50, 0.62)	< 0.00	24,957.96
WIN_lag2^a^ (m/s)	− 0.70 (− 0.81, − 0.59)	0.50 (0.44, 0.55)	< 0.00	24,915.83
WIN_lag3 (m/s)	− 0.68 (− 0.79, − 0.57)	0.50 (0.45, 0.56)	< 0.00	24,925.07
PRE_lag0 (dm)	0.20 (0.15, 0.24)	1.22 (1.17, 1.28)	< 0.00	24,991.31
PRE_lag1 (dm)	0.45 (0.40, 0.49)	1.56 (1.49, 1.64)	< 0.00	24,711.30
PRE_lag2^a^ (dm)	0.55 (0.45, 0.55)	1.65 (1.57, 1.73)	< 0.00	24,648.56
PRE_lag3 (dm)	0.37 (0.32, 0.42)	1.44 (1.38, 1.52)	< 0.00	24,844.05
Socioeconomic				
Dep (1%)	0.06 (0.05, 0.06)	1.06 (1.05, 1.06)	< 0.00	24,790.29
Dep_Old^a^ (1%)	0.07 (0.05, 0.08)	1.07 (1.05, 1.08)	< 0.00	24,999.00
Dep_Yong (1%)	0.11 (0.10, 0.12)	1.11 (1.10, 1.13)	< 0.00	24,652.98
Pop_den^a^ (10^3^ persons/km^2^)	− 0.02 (− 0.03, − 0.01)	0.98 (0.97, 0.99)	< 0.00	25,061.71
GDP (10^4^ RMB/person)	− 0.02 (− 0.03, − 0.01)	0.98 (0.97, 0.99)	< 0.00	25,062.30
Topographical/land cover				
Slope (°)	0.04 (0.03, 0.05)	1.04 (1.03, 1.05)	< 0.00	25,030.43
Dem (100 m)	− 0.03 (− 0.06, − 0.01)	0.97 (0.94, 0.99)	0.01	25,062.16
NDVI^a^	1.74 (1.44, 2.04)	5.71 (4.24, 7.71)	< 0.00	24,949.65
Cropland (10%)	− 0.08 (− 11.26, − 0.05)	0.92 (0.89, 0.96)	< 0.00	25,049.58
Forest (10%)	0.08 (0.06, 0.09)	1.08 (1.06, 1.10)	< 0.00	24,986.46
Shrubland (0.1%)^a^	0.88 (0.78, 0.98)	2.41 (2.18, 2.67)	< 0.00	24,684.74
Grassland (1%)	− 0.15 (− 0.63, 0.34)	0.86 (0.53, 1.39)	0.54	25,069.81
Water bodies (1%)^a^	− 0.10 (− 0.11, 0.92)	0.91 (0.89, 0.92)	< 0.00	24,960.07
Barren (1%)^a^	− 0.27 (− 0.45, − 0.09)	0.76 (0.63, 0.92)	< 0.00	25,062.61

*IRR* Incidence rate ratio,* AIC* Akaike information criterion, *lagk* lag period where *k* is number of months; for other abbreviations, see Table 1

^a^Variable included in the multivariable regression model

The final multivariate negative binomial regression showed that six factors were positively and two factors negatively associated with scrub typhus cases (Table 3). The annual mean normalized difference vegetation index (NDVI) was most strongly correlated with the number of scrub typhus cases (IRR = 9.15, 95% CI 5.02–16.68, *P* < 0.001). The model indicated that each 0.1% rise in shrubland led to a 75.0% increase in monthly scrub typhus cases (IRR = 1.75, 95% CI 1.60–1.90, *P* < 0.001), each 1% rise in barren land led to a 37.0% increase in the monthly number of cases (IRR = 1.37, 95% CI 1.14–1.64, *P* < 0.001), each 1 kPa increase in the monthly average atmospheric pressure (PRS) in the previous 3 months led to a 30.0% increase in the monthly number of cases (IRR = 1.30, 95% CI 1.26–1.34, *P* < 0.001), and each 1 ℃ increase in TEM in the previous month led to a 18.0% increase in the monthly number of cases (IRR = 1.18, 95% CI 1.17–1.19, *P* < 0.001). Each unit rise in mean wind speed (WIN) in the previous 2 months corresponded to a decrease of 40.0% in the monthly number of cases (IRR = 0.60, 95% CI 0.53–0.67, *P* < 0.001), and each 1% increase in water bodies corresponded to a decrease of 4.0% in the monthly number of cases (IRR = 0.96, 95% CI 0.94–0.98, *P* < 0.001). The relationships between the variables and scrub typhus cases are shown in Table 3.

**Table 3:** Final multivariate negative binomial regression model for panel data, showing coefficients, IRR, 95% confidence intervals (*CI*s) and *P*-values

Variable (unit)	Coefficient (95% CI)	IRR (95% CI)	*P*-value
Intercept	− 30.25 (− 33.26, − 27.24)		< 0.00
PRS_lag3 (kPa)	0.26 (0.23, 0.29)	1.30 (1.26, 1.34)	< 0.00
TEM_lag1 (℃)	0.17 (0.16, 0.17)	1.18 (1.17, 1.19)	< 0.00
WIN_lag2	− 0.52 (− 0.63, − 4.00)	0.60 (0.53, 0.67)	< 0.00
Pop_den (10^3^ persons/km^2^)	0.05 (0.03, 0.06)	1.05 (1.03, 1.06)	< 0.00
NDVI	2.21 (1.61, 2.81)	9.15 (5.02, 16.68)	< 0.00
Shrubland (0.1%)	0.56 (0.47, 0.65)	1.75 (1.60, 1.90)	< 0.00
Water bodies (1%)	− 0.04 (− 0.07, − 0.02)	0.96 (0.94, 0.98)	< 0.00
Barren (1%)	0.31 (0.13, 0.50)	1.37 (1.14, 1.64)	< 0.00

For other abbreviations, see Table 1

### Model evaluation

**Fig. 3:**
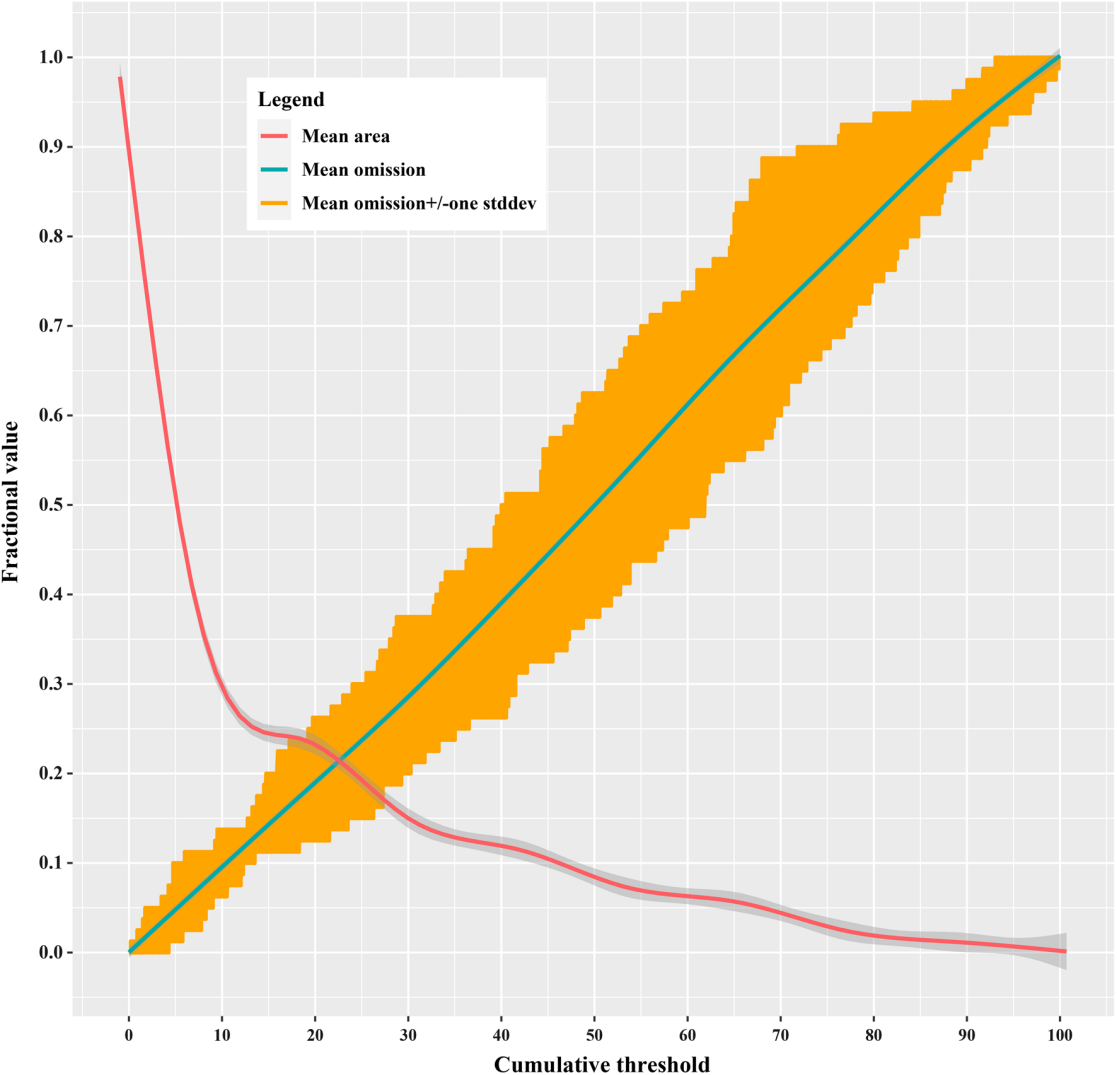
Average omission rate and predicted area for scrub typhus in Fujian Province

**Fig. 4:**
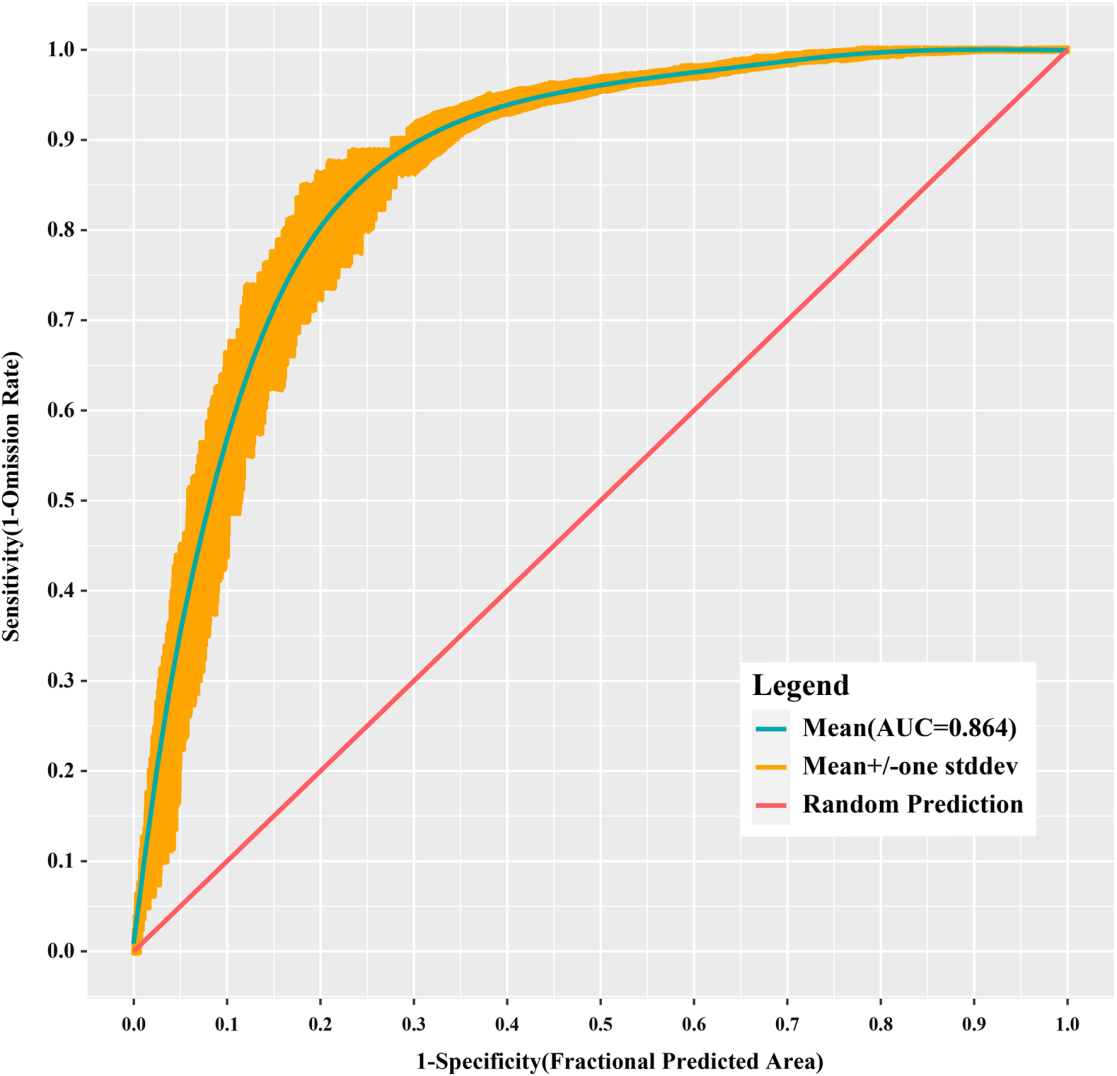
Receiver operating characteristic curve of the maximum entropy model for scrub typhus in Fujian Province

Figure 3 shows the omission rate and predicted area as a function of the cumulative threshold. The mean omission rate of the test data was close to the predicted omission, and the training model was considered to be statistically significant. The average AUC for the 10 replicate runs in the training model was 0.864 with a SD of 0.015, indicating that the MaxEnt model prediction is sensitive and accurate, and the prediction capability of the model is robust (Fig. 4). The model evaluation results of each individual run are shown in Additional file 1: Table S1.

### Key predictive variables for scrub typhus

**Fig. 5:**
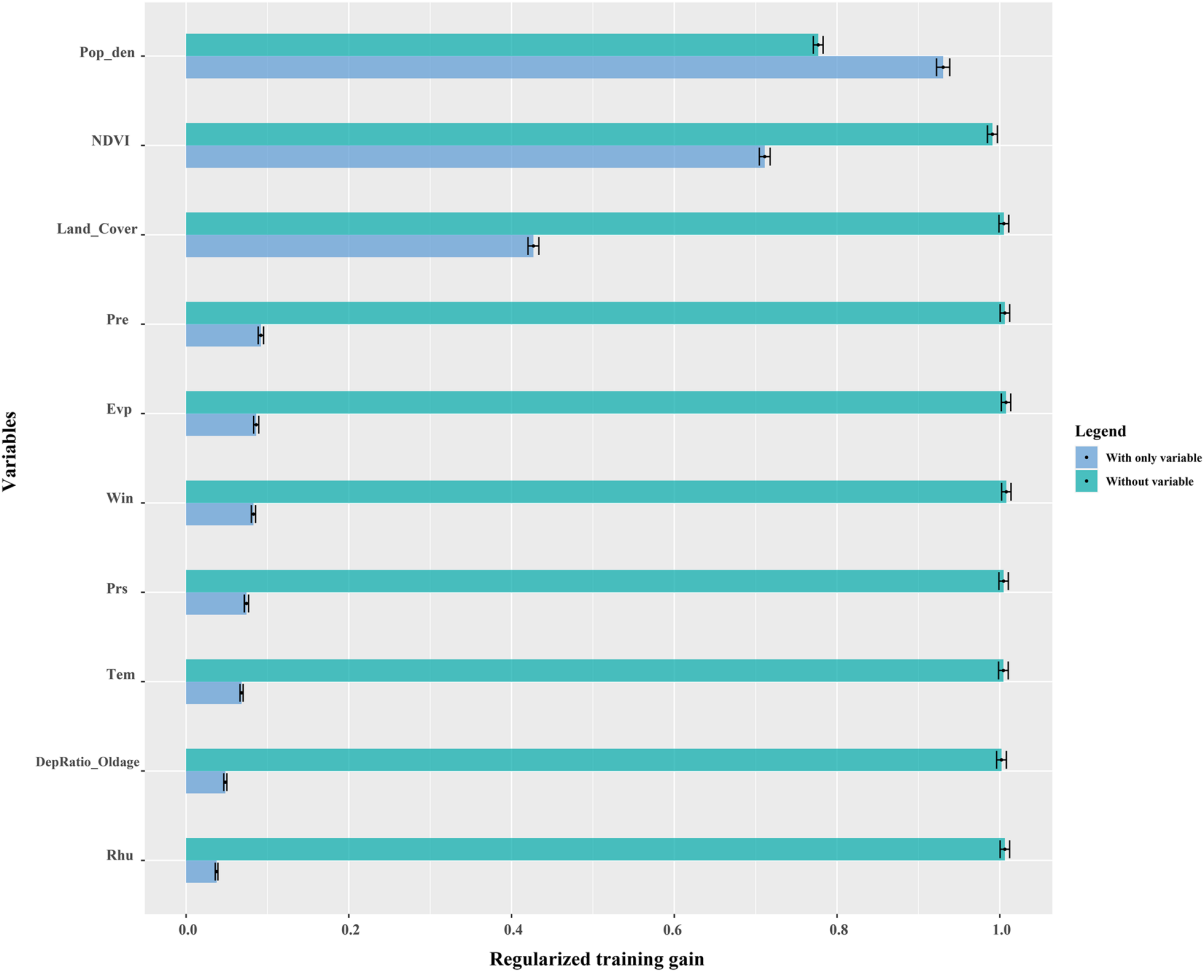
Jackknife results showing the relative contribution of predictor variables to the prediction of relative risk of scrub typhus in Fujian Province

**Fig. 6:**
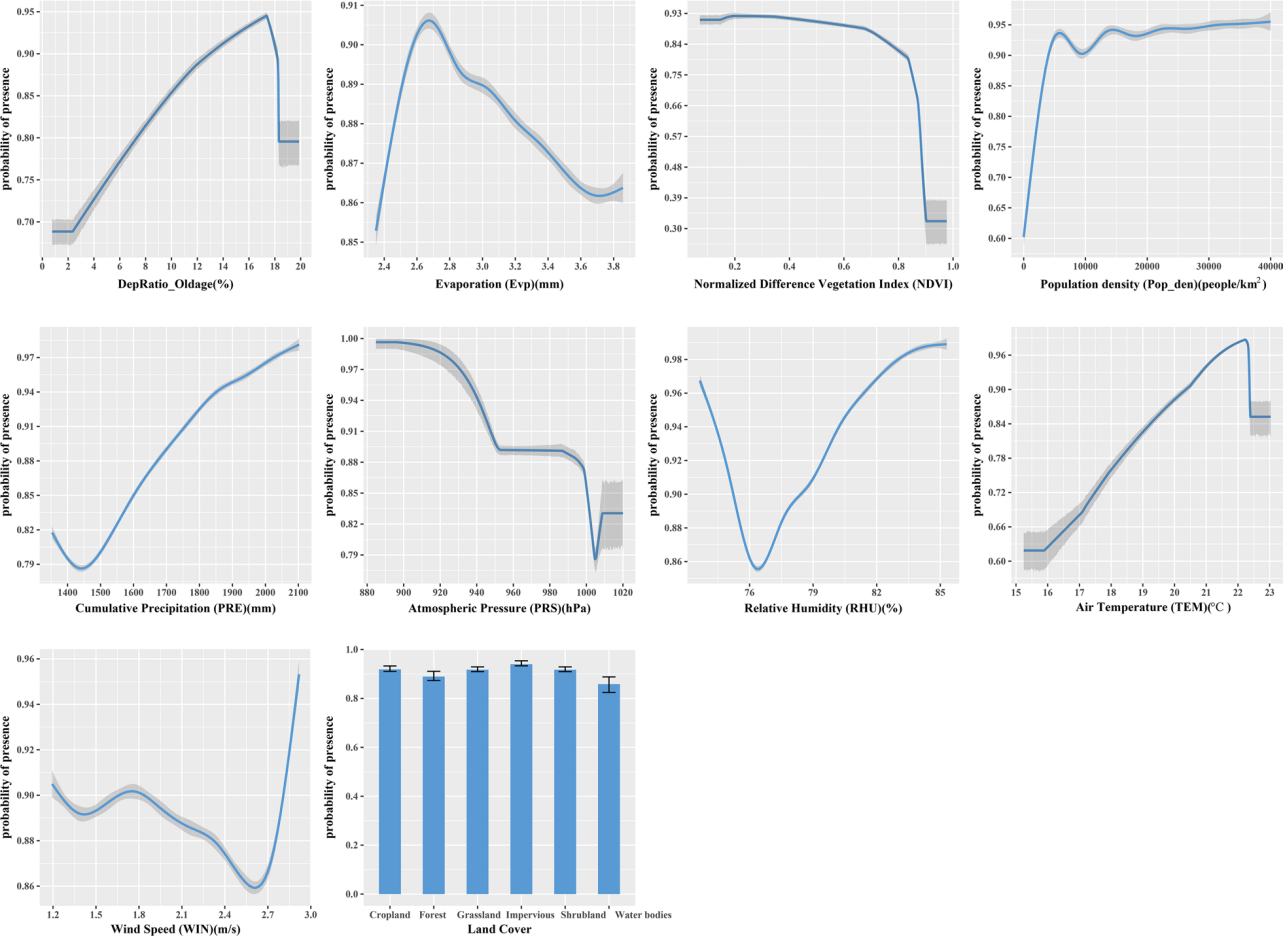
Response curves for probability of presence of scrub typhus. The curves show the mean response of the 10 replicate MaxEnt runs (blue) and the mean ± SD (gray). *x*-axis shows the value of each predictor variable, and the *y-*axis the probability of scrub typhus occurrence

The variables with a high relative contribution to the MaxEnt model, accounting for 81.5%, 7.4% and 5.1%, respectively, and a total contribution of 94%, were Population Density (Pop_den), NDVI and land cover. The jackknife test indicated that, when used in isolation, Pop_den was the variable with the highest gain (Fig. 5). The response curve plots (Fig. 6) indicated that Pop_den had a positive impact on the occurrence of scrub typhus, and that the optimum range was above 15,000. Although the overall response for NDVI was negative, it was better at indicating suitable environments for scrub typhus occurrence, with response values of approximately 0.1–0.7. Suitability was highest at around 884 hpa for PRS, then gradually decreased at higher values. When WIN was lower than 2.6 m/s, it had a negative relationship with the occurrence of scrub typhus, but when it was above 2.6 m/s, the response was positive. Occurrence of scrub typhus was highest at a TEM of approximately 22℃. There was an overall positive relationship between scrub typhus occurrence and Dep_Old and mean relative humidity, while with EVP the overall response was negative. Among the land cover variables, the probability of scrub typhus occurrence was highest for impervious surfaces and cropland.

### Environmental suitability for scrub typhus

**Fig. 7:**
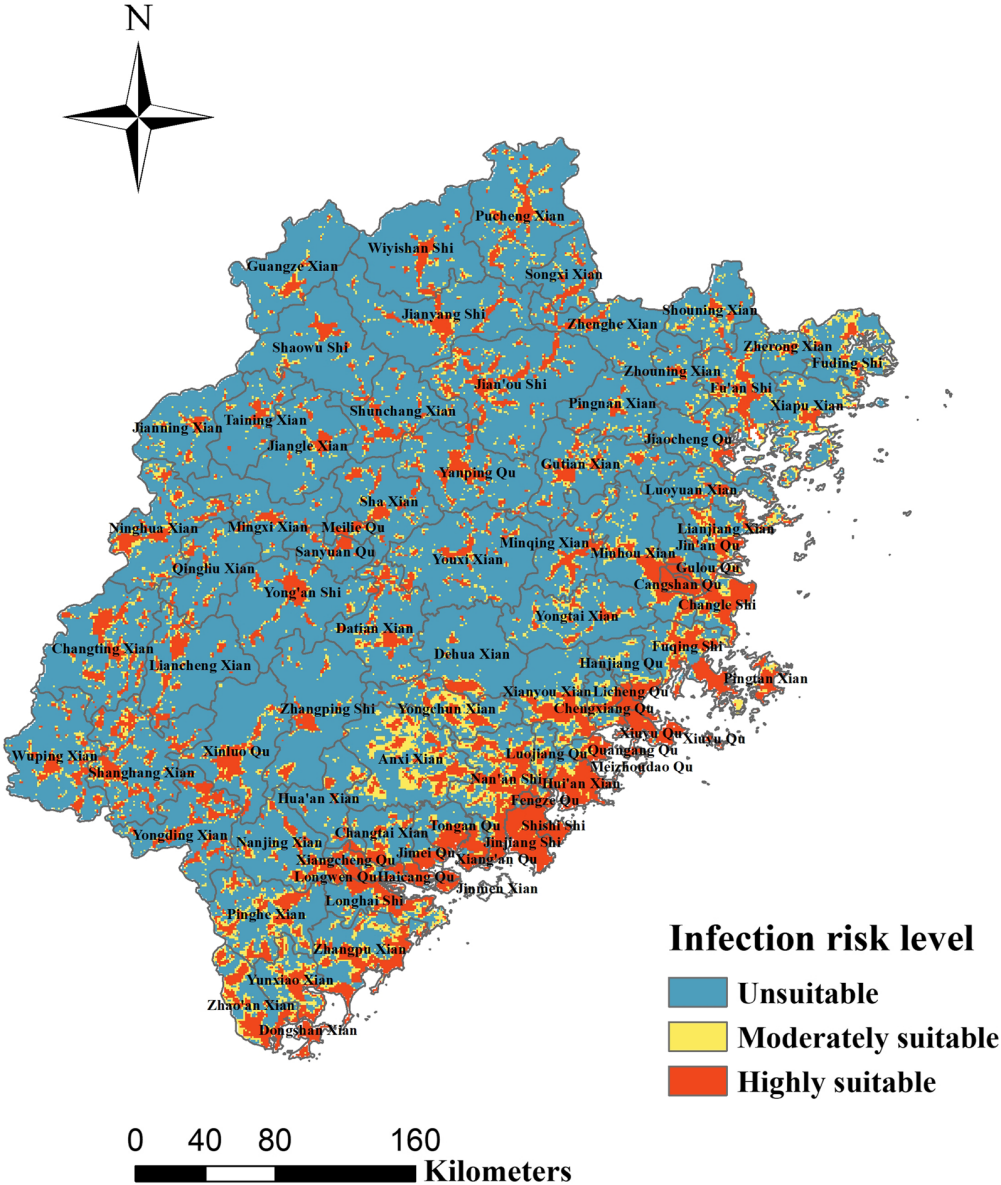
Environmental suitability maps for scrub typhus in Fujian Province

Figure 7 shows the environmentally suitable areas for the distribution of scrub typhus in Fujian Province, with diverse scales and zones with different levels of infection risk in different counties. The MaxEnt model indicated that 16.14% of the area was highly suitable for the distribution of scrub typhus, 9.42% moderately suitable and 74.43% unsuitable. Additional file 1: Table S2 shows the areas and populations at different levels of infection risk at the county level. The highly suitable areas are mainly distributed in eastern coastal regions of Fujian Province, especially in cities such as Fuzhou, Xiamen and Quanzhou, with relatively sparse and sporadic distributions in the remaining regions. The moderately suitable areas were mainly distributed in Anxi County and its adjacent districts, as well as in the southern parts of Fujian Province; the rest were mainly located in smaller areas at the boundaries with the highly suitable regions. The unsuitable areas were widely distributed throughout the whole province, but most prevalent in the central high-altitude areas.

In sum, the results showed that most of the population, i.e. 81.63%, live in highly suitable areas for the occurrence of scrub typhus, whereas 5.96% live in moderately suitable areas, and 12.41% in unsuitable areas.

## Discussion

A negative binomial regression model for panel data was used to identify various risk factors associated with the occurrence of scrub typhus. Scrub typhus cases were positively correlated with NDVI, shrubland, barren land, PRS in the previous 3 months, and TEM in the previous 1 month. In contrast, cases were negatively correlated with an increase in WIN and area covered by water bodies. We mapped the areas in Fujian Province with suitable environmental conditions for scrub typhus, and estimated the size of the populations at different levels of infection risk, by using the MaxEnt model, which had a robust prediction capability, with an average AUC as high as 0.864. NDVI and land cover were the most important variables indicating the probability of the presence of scrub typhus in Fujian Province. Furthermore, the eastern coastal region of Fujian Province, in which the majority of the province’s inhabitants live, had a highly suitable environment for the occurrence of scrub typhus, which indicates that its inhabitants may have an elevated risk of infection in the future.

Scrub typhus cases were strongly correlated with NDVI and shrubland, which were key predictive variables. In general, chiggers and hosts were abundant in secondary vegetation, which grows following anthropogenic or natural disturbance. This type of vegetation can serve as a point from which chiggers can attach to passing hosts [28, 29]. One study reported that a positive correlation between scrub typhus and rodent density led to a high incidence of scrub typhus [30]. Other studies demonstrated that people living in houses surrounded by bushes, shrubs or grasses had an increased risk of developing scrub typhus [31, 32]. In addition, the formation of new foci is often related to similar meteorological conditions, and types of vegetation which provide suitable habitats for hosts and chiggers [10]. Mosaics comprising cropland and other types of vegetation had the most significant correlation with the risk of scrub typhus, possibly because they provide sufficient food for the growth and reproduction of hosts [33]. A Taiwanese study showed that the widespread abandonment of farmland in remote areas, a consequence of industrialization and rural-to-urban migration, provided the conditions for the invasion of exotic plants, which increased the public’s risk of the disease [34]. All of the patterns discussed above show an important link between scrub typhus and NDVI, and that the disease is positively related vegetation cover [28]. The proportion of land impervious to water penetration is also deemed an important predictor of the probability of scrub typhus. A survival analysis undertaken for mainland China showed that intersection of an area by a freeway was a risk factor associated with the spatial expansion of scrub typhus, one possible explanation for which was an increase in trade of agricultural products and livestock along the highway [35]. The effect of impervious surfaces on the occurrence of scrub typhus may also be closely related to urbanization and urban population density. In sum, different types of land cover are important drivers of scrub typhus emergence.

Temperature plays a significant role in the occurrence of scrub typhus, as it influences the activities of rodents, mites and humans, and the average temperature was positively correlated with scrub typhus cases. Increases in the reproduction and abundance of rodent hosts and proliferation of the pathogen with temperature also lead to an increase in the activity of chiggers [20, 36]. Thus, the greenhouse effect and global warming may also increase the probability of occurrence of scrub typhus. *Leptotrombidium deliense* is the major vector of scrub typhus south of the Yangtze River in China, and typically causes scrub typhus in the summer as people are more inclined to go outside during the summer holidays in this region [35]. In our previous study [9], scrub typhus cases in Fujian Province had two seasonal peaks, one in June-July and one in September–October, with the highest number of cases in the former. The seasonal peak of *Leptotrombidium deliense* coincided with the peak incidence of scrub typhus, which provided support for the assumption that temperature has a positive effect on scrub typhus. However, a negative correlation between the incidence of scrub typhus and temperature, possibly due to differences in dominant strains and meteorological conditions between regions, has been reported [30].

In contrast to our findings, a study undertaken in Guangzhou City showed a negative association between atmospheric pressure and scrub typhus, which may indicate that high atmospheric pressure adversely affects the survival of mites [12]. A possible explanation for this is lower temperature and humidity at higher elevations, and low atmospheric pressure at high altitude. This explanation corresponds with our MaxEnt model results that showed that the most unsuitable area for the occurrence of scrub typhus is located at high altitude in central Fujian Province. There was also a trend showing a decrease in the proportion of vegetation with increasing altitude. None of these conditions are beneficial to the survival of chiggers and their hosts, and thus result in a low incidence of scrub typhus. Atmospheric pressure had a strong positive correlation with mean sunshine duration in the present study. Longer periods of sunshine can also increase the risk of scrub typhus infection [12], which may also explain our results. In addition, the response curve of the MaxEnt model showed an overall negative relationship between atmospheric pressure and the occurrence of scrub typhus, which also deserves attention.

Population density was an important influencing factor and predictive variable for scrub typhus. Changes in land use, animal populations and climate caused by anthropogenic activities, and increasing human populations, have contributed to the emergence of zoonotic diseases [37]. Urban population growth is accompanied by highly heterogeneous environmental and socioeconomic conditions, which may impact the demographic dynamics of vector-borne diseases [38]. Proximity to urban areas was the most important risk factor affecting the occurrence and spread of scrub typhus in Nepal, and may have been related to the urban population growth [27]. The coastal cities of Fujian Province have thriving economies and high population densities. The extensive construction of parks and greening of streets for the creation of more environmentally sustainable societies in these cities, which are undergoing accelerating urbanization, also lead to an increase in suitable habitats for mites and rodents. At the same time, trade in coastal cities such as Xiamen and Fuzhou City is booming, as is tourism, especially in the summer. The good transport system in this area enabling large numbers of tourists to travel and congregate may indirectly help the spread of the pathogen. In general, coastal areas have a higher relative humidity, and studies have shown a positive relationship between relative humidity and scrub typhus, probably because the former is beneficial to chigger survival [12]. However, there are few studies on the effects of population density on scrub typhus occurrence, and a potential relationship between them needs to be explored in future research.

Few studies have explored the relationship between wind velocity and scrub typhus. Those that have generally indicate that they are negatively correlated. This is consistent with our results, but the underlying mechanism associated with this relationship is not clear. Windy weather is often accompanied by rain, so people are less likely to go outside when it is windy, which in turn leads to a reduction, for example, in the number of people occupied in farming or other outdoor activities who come in contact with vegetation [36]. It is also possible that high wind speeds are not conducive to the survival and development of mites, as their eggs may be blown far away from suitable habitat for the development of mites to the adult stage. Windy weather may also influence the activity of rodents. Thus all of these possible effects of windy weather may lead to a reduction in scrub typhus infections. A Korean study showed that scrub typhus was strongly correlated with average wind speed [39], and that the latter had an effect on chigger reproduction, although the effect of wind speed on scrub typhus epidemiology and ecology was not examined. Hence, the relationship between wind speed and the ecology and life cycle of chiggers and mechanisms of scrub typhus infection need to be examined in future research.

The most suitable environments for scrub typhus in Fujian Province were mainly distributed in the coastal region, and especially the cities of Xiamen, Fuzhou, Quanzhou, and Putian. Possible reasons for this could be the large human populations, suitable climatic conditions and land cover types of the coastal areas. In addition, most of the human population was concentrated in areas of Fujian Province that were found to be highly suitable for scrub typhus. Although the relative proportion of these highly suitable areas is low, the proportion of the population most at risk of scrub typhus transmission is high in these areas [27]. However, it is important to recognize that, even in the most conducive settings, not everyone will contract scrub typhus [40].

The strengths of our study included analysis of the simultaneous effects of meteorological, socioeconomic and land cover variables on the occurrence of scrub typhus, which may have mitigated the impact of confounding factors. Secondly, few previous studies have examined possible lags in the effects of meteorological factors on scrub typhus, which may arise due to the incubation period of the mites. Thirdly, we used a MaxEnt model that combined an integrated database comprising different factors to predict the emergence of scrub typhus, and mapped the suitability of different environments for the disease. These findings may serve to increase awareness of scrub typhus, and are useful for public health institutes undertaking surveillance and devising and implementing targeted prevention and control strategies for areas at high risk of occurrence of the disease. However, there are also potential limitations in our study. Firstly, as all of the data were collected from passive monitoring systems, under-reporting was inevitable. Secondly, as the socioeconomic variables did not significantly change for any county of Fujian Province during our study, they did not have a considerable impact on our results, but they may show a change over a longer period of time. Thirdly, as this was an ecological study, ecological fallacies may have influenced the findings.

## Conclusions

Scrub typhus is an important public health concern in Fujian Province, China. We used ecological niche modeling to predict the potential distribution of scrub typhus based on occurrence data and different risk factors for the disease in Fujian Province. Our results may be of use to public health authorities for the implementation of effective surveillance and control strategies for specific risk factors of scrub typhus. The risk map may help public health authorities in Fujian Province to predict which populations are at high risk of scrub typhus, and also predict the spread of this disease. The findings of this study can also be extrapolated to similar areas endemic for scrub typhus for the implementation of targeted measures.

## Supplementary Information

**Additional file 1. Table S1**: Model evaluation results of each single run. **Table S2**: Estimated human population exposed to different levels of scrub typhus transmission suitability in Fujian Province at the county level.

## Data Availability

The datasets used and/or analyzed during the current study are available from the corresponding author on reasonable request.
