# Supplementary material for: Using ecological niche modeling to predict the potential distribution of scrub typhus in Fujian Province, China
Source: Parasit Vectors. 2023 Jan 31;16:44. doi: 10.1186/s13071-023-05668-6 (PMC9887782; doi:10.1186/s13071-023-05668-6)
Supplement: Supplementary file 1 — Additional file 1. Table S1: Model evaluation results of each single run. Table S2: Estimated human population exposed to different levels of scrub typhus transmission suitability in Fujian Province at the county level. [file 13071_2023_5668_MOESM1_ESM.docx]

| **Table S1** Model evaluation results of each single run | | | | | |
| --- | --- | --- | --- | --- | --- |
| Run | Training AUC | Testing AUC | Training omission rate^a^ | Testing omission rate | *P* value |
| 1 | 0.871 | 0.857 | 0.039 | 0.050 | <0.0001 |
| 2 | 0.871 | 0.853 | 0.031 | 0.037 | <0.0001 |
| 3 | 0.872 | 0.844 | 0.032 | 0.025 | <0.0001 |
| 4 | 0.870 | 0.866 | 0.024 | 0.050 | <0.0001 |
| 5 | 0.870 | 0.885 | 0.036 | 0.000 | <0.0001 |
| 6 | 0.871 | 0.850 | 0.028 | 0.050 | <0.0001 |
| 7 | 0.871 | 0.857 | 0.035 | 0.075 | <0.0001 |
| 8 | 0.870 | 0.856 | 0.036 | 0.050 | <0.0001 |
| 9 | 0.868 | 0.886 | 0.036 | 0.013 | <0.0001 |
| 10 | 0.869 | 0.885 | 0.036 | 0.037 | <0.0001 |
| ^a^Balance training omission,predicted area and the threshold value | | | | | |

| **Table S2**.Estimated human population exposed at different levels of scrub typhus transmission suitability in Fujian province at county level. | | | | | |
| --- | --- | --- | --- | --- | --- |
| District/County | Class of Suitability | Area(Km^2^) | Population | Area (%) | Population (%) |
| Total | Unsuitable | 812,336.70 | 5,522,622 | 74.43 | 12.41 |
|  | Moderately suitable | 102,864.51 | 2,649,453 | 9.42 | 5.96 |
|  | Highly suitable | 176,201.97 | 36,316,693 | 16.14 | 81.63 |
| Anxi Xian | Unsuitable | 15,752.06 | 173,983 | 1.44 | 0.39 |
|  | Moderately suitable | 6,810.83 | 244,889 | 0.62 | 0.55 |
|  | Highly suitable | 3,615.23 | 799,090 | 0.33 | 1.8 |
| Cangshan Qu | Unsuitable | 24.21 | 119 | 0.00 | 0.00 |
|  | Moderately suitable | 56.49 | 2,041 | 0.01 | 0.00 |
|  | Highly suitable | 1,678.50 | 1,321,071 | 0.15 | 2.97 |
| Chengxiang Qu | Unsuitable | 2,493.54 | 42,090 | 0.23 | 0.09 |
|  | Moderately suitable | 911.88 | 43,376 | 0.08 | 0.1 |
|  | Highly suitable | 1,210.46 | 380,650 | 0.11 | 0.86 |
| Datian Xian | Unsuitable | 15,211.39 | 81,490 | 1.39 | 0.18 |
|  | Moderately suitable | 1,985.15 | 41,681 | 0.18 | 0.09 |
|  | Highly suitable | 2,784.05 | 229,776 | 0.26 | 0.52 |
| Dehua Xian | Unsuitable | 18,051.93 | 64,095 | 1.65 | 0.14 |
|  | Moderately suitable | 847.32 | 22,907 | 0.08 | 0.05 |
|  | Highly suitable | 1,283.08 | 258,463 | 0.12 | 0.58 |
| Dongshan Xian | Unsuitable | 492.25 | 49,985 | 0.05 | 0.11 |
|  | Moderately suitable | 40.35 | 892 | 0.00 | 0.00 |
|  | Highly suitable | 1,097.48 | 149,582 | 0.1 | 0.34 |
| Fengze Qu | Unsuitable | 161.39 | 11,238 | 0.01 | 0.03 |
|  | Moderately suitable | 129.12 | 9,029 | 0.01 | 0.02 |
|  | Highly suitable | 613.30 | 587,757 | 0.06 | 1.32 |
| Fu'an Shi | Unsuitable | 10,974.80 | 84,657 | 1.01 | 0.19 |
|  | Moderately suitable | 2,194.96 | 51,737 | 0.20 | 0.12 |
|  | Highly suitable | 3,171.39 | 466,292 | 0.29 | 1.05 |
| Fuding Shi | Unsuitable | 8,949.30 | 139,247 | 0.82 | 0.31 |
|  | Moderately suitable | 2,654.93 | 90,449 | 0.24 | 0.20 |
|  | Highly suitable | 1,210.46 | 255,631 | 0.11 | 0.57 |
| Fuqing Shi | Unsuitable | 6,205.60 | 166,583 | 0.57 | 0.37 |
|  | Moderately suitable | 1,743.06 | 63,599 | 0.16 | 0.14 |
|  | Highly suitable | 4,680.43 | 1,142,900 | 0.43 | 2.57 |
| Gutian Xian | Unsuitable | 17,002.87 | 87,534 | 1.56 | 0.20 |
|  | Moderately suitable | 1,904.45 | 31,258 | 0.17 | 0.07 |
|  | Highly suitable | 2,582.31 | 229,050 | 0.24 | 0.51 |
| Gulou Qu | Unsuitable | 0.00 | 0 | 0 | 0 |
|  | Moderately suitable | 0.00 | 0 | 0 | 0 |
|  | Highly suitable | 355.07 | 924,800 | 0.03 | 2.08 |
| Guangze Xian | Unsuitable | 19,004.15 | 59,081 | 1.74 | 0.13 |
|  | Moderately suitable | 798.90 | 11,578 | 0.07 | 0.03 |
|  | Highly suitable | 960.29 | 65,922 | 0.09 | 0.15 |
| Haicang Qu | Unsuitable | 395.42 | 31,914 | 0.04 | 0.07 |
|  | Moderately suitable | 104.91 | 11,533 | 0.01 | 0.03 |
|  | Highly suitable | 919.95 | 548,091 | 0.08 | 1.23 |
| Hanjiang Qu | Unsuitable | 4,519.03 | 51,306 | 0.41 | 0.12 |
|  | Moderately suitable | 863.46 | 27,995 | 0.08 | 0.06 |
|  | Highly suitable | 1,121.69 | 363,299 | 0.1 | 0.82 |
| Huli Qu | Unsuitable | 112.98 | 96,448 | 0.01 | 0.22 |
|  | Moderately suitable | 0.00 | 0 | 0 | 0 |
|  | Highly suitable | 379.28 | 892,632 | 0.03 | 2.01 |
| Hua'an Xian | Unsuitable | 9,748.20 | 54,552 | 0.89 | 0.12 |
|  | Moderately suitable | 952.23 | 22,579 | 0.09 | 0.05 |
|  | Highly suitable | 1,089.41 | 92,056 | 0.10 | 0.21 |
| Hui'an Xian | Unsuitable | 1,129.76 | 94,834 | 0.10 | 0.21 |
|  | Moderately suitable | 581.02 | 24,897 | 0.05 | 0.06 |
|  | Highly suitable | 4,018.71 | 867,925 | 0.37 | 1.95 |
| Jimei Qu | Unsuitable | 1,016.78 | 73,725 | 0.09 | 0.17 |
|  | Moderately suitable | 177.53 | 10,315 | 0.02 | 0.02 |
|  | Highly suitable | 1,121.69 | 878,206 | 0.10 | 1.97 |
| Jianning Xian | Unsuitable | 13,904.10 | 66,403 | 1.27 | 0.15 |
|  | Moderately suitable | 1,040.99 | 14,031 | 0.10 | 0.03 |
|  | Highly suitable | 928.02 | 48,193 | 0.09 | 0.11 |
| Jian'ou Shi | Unsuitable | 30,600.32 | 127,606 | 2.80 | 0.29 |
|  | Moderately suitable | 2,816.33 | 40,655 | 0.26 | 0.09 |
|  | Highly suitable | 5,027.43 | 318,522 | 0.46 | 0.72 |
| Jianyang Shi | Unsuitable | 26363.72 | 80,583 | 2.42 | 0.18 |
|  | Moderately suitable | 1678.50 | 20,982 | 0.15 | 0.05 |
|  | Highly suitable | 2767.91 | 197,469 | 0.25 | 0.44 |
| Jiangle Xian | Unsuitable | 18,164.90 | 53,283 | 1.66 | 0.12 |
|  | Moderately suitable | 960.29 | 14,047 | 0.09 | 0.03 |
|  | Highly suitable | 1,387.99 | 93,645 | 0.13 | 0.21 |
| Jiaocheng Qu | Unsuitable | 9,732.06 | 72,137 | 0.89 | 0.16 |
|  | Moderately suitable | 1,040.99 | 24,986 | 0.10 | 0.06 |
|  | Highly suitable | 1,404.13 | 303,085 | 0.13 | 0.68 |
| Jin'an Qu | Unsuitable | 4,212.39 | 20,300 | 0.39 | 0.05 |
|  | Moderately suitable | 290.51 | 17,698 | 0.03 | 0.04 |
|  | Highly suitable | 621.37 | 511,054 | 0.06 | 1.15 |
| Jinjiang Shi | Unsuitable | 338.93 | 73,007 | 0.03 | 0.16 |
|  | Moderately suitable | 137.18 | 12,435 | 0.01 | 0.03 |
|  | Highly suitable | 5,334.07 | 2,406,869 | 0.49 | 5.41 |
| Licheng Qu | Unsuitable | 0.00 | 0 | 0 | 0 |
|  | Moderately suitable | 8.07 | 1,907 | 0.00 | 0.00 |
|  | Highly suitable | 451.90 | 476,878 | 0.04 | 1.07 |
| Licheng Qu | Unsuitable | 435.76 | 11,520 | 0.04 | 0.03 |
|  | Moderately suitable | 153.32 | 7,380 | 0.01 | 0.02 |
|  | Highly suitable | 1,420.27 | 539,328 | 0.13 | 1.21 |
| Liancheng Xian | Unsuitable | 19,447.99 | 51,332 | 1.78 | 0.12 |
|  | Moderately suitable | 1,226.59 | 17,568 | 0.11 | 0.04 |
|  | Highly suitable | 2,638.79 | 190,536 | 0.24 | 0.43 |
| Lianjiang Xian | Unsuitable | 5,075.84 | 144,194 | 0.47 | 0.32 |
|  | Moderately suitable | 1,767.27 | 53,529 | 0.16 | 0.12 |
|  | Highly suitable | 2,759.84 | 468,937 | 0.25 | 1.05 |
| Longhai Shi | Unsuitable | 5245.31 | 101,090 | 0.48 | 0.23 |
|  | Moderately suitable | 1597.80 | 49,619 | 0.15 | 0.11 |
|  | Highly suitable | 3954.15 | 838,541 | 0.36 | 1.88 |
| Longwen Qu | Unsuitable | 234.02 | 2,442 | 0.02 | 0.01 |
|  | Moderately suitable | 201.74 | 4,642 | 0.02 | 0.01 |
|  | Highly suitable | 589.09 | 116,241 | 0.05 | 0.26 |
| Luoyuan Xian | Unsuitable | 7,440.27 | 54,991 | 0.68 | 0.12 |
|  | Moderately suitable | 879.60 | 19,031 | 0.08 | 0.04 |
|  | Highly suitable | 944.16 | 150,640 | 0.09 | 0.34 |
| Luojiang Qu | Unsuitable | 1,847.96 | 27,391 | 0.17 | 0.06 |
|  | Moderately suitable | 798.90 | 40,471 | 0.07 | 0.09 |
|  | Highly suitable | 750.48 | 142,130 | 0.07 | 0.32 |
| Mawei Qu | Unsuitable | 855.39 | 39,368 | 0.08 | 0.09 |
|  | Moderately suitable | 242.09 | 14,789 | 0.02 | 0.03 |
|  | Highly suitable | 532.60 | 169,724 | 0.05 | 0.38 |
| Meilie Qu | Unsuitable | 2,445.12 | 7,578 | 0.22 | 0.02 |
|  | Moderately suitable | 242.09 | 4,363 | 0.02 | 0.01 |
|  | Highly suitable | 669.79 | 149,570 | 0.06 | 0.34 |
| Meizhoudao Qu | Unsuitable | 48.42 | 6,890 | 0.00 | 0.02 |
|  | Moderately suitable | 0.00 | 0 | 0 | 0 |
|  | Highly suitable | 32.28 | 5,312 | 0.00 | 0.01 |
| Meizhou Bay North Shore Economic Development Qu | Unsuitable | 201.74 | 22,985 | 0.02 | 0.05 |
|  | Moderately suitable | 24.21 | 715 | 0.00 | 0.00 |
|  | Highly suitable | 298.58 | 39,541 | 0.03 | 0.09 |
| Minhou Xian | Unsuitable | 13,508.68 | 83,339 | 1.24 | 0.19 |
|  | Moderately suitable | 2,170.75 | 66,665 | 0.20 | 0.15 |
|  | Highly suitable | 3,373.14 | 669,315 | 0.31 | 1.50 |
| Minqing Xian | Unsuitable | 10,353.43 | 56,542 | 0.95 | 0.13 |
|  | Moderately suitable | 1,162.04 | 26,387 | 0.11 | 0.06 |
|  | Highly suitable | 2,041.63 | 215,900 | 0.19 | 0.49 |
| Mingxi Xian | Unsuitable | 14,073.56 | 46,643 | 1.29 | 0.10 |
|  | Moderately suitable | 597.16 | 8,759 | 0.05 | 0.02 |
|  | Highly suitable | 911.88 | 56,895 | 0.08 | 0.13 |
| Nan'an Shi | Unsuitable | 6,625.23 | 122,461 | 0.61 | 0.28 |
|  | Moderately suitable | 3,994.50 | 181,644 | 0.37 | 0.41 |
|  | Highly suitable | 6,939.94 | 1,444,346 | 0.64 | 3.25 |
| Nanjing Xian | Unsuitable | 12,814.69 | 87,175 | 1.17 | 0.20 |
|  | Moderately suitable | 1,864.10 | 42,044 | 0.17 | 0.09 |
|  | Highly suitable | 2,945.44 | 259,234 | 0.27 | 0.58 |
| Ninghua Xian | Unsuitable | 15,388.92 | 77,311 | 1.41 | 0.17 |
|  | Moderately suitable | 2,388.63 | 33,005 | 0.22 | 0.07 |
|  | Highly suitable | 3,405.41 | 171,251 | 0.31 | 0.38 |
| Pinghe Xian | Unsuitable | 12,629.09 | 102,242 | 1.16 | 0.23 |
|  | Moderately suitable | 3,695.92 | 84,596 | 0.34 | 0.19 |
|  | Highly suitable | 4,042.92 | 392,415 | 0.37 | 0.88 |
| Pingtan Xian | Unsuitable | 895.74 | 82,214 | 0.08 | 0.18 |
|  | Moderately suitable | 290.51 | 9,715 | 0.03 | 0.02 |
|  | Highly suitable | 1,275.01 | 295,337 | 0.12 | 0.66 |
| Pingnan Xian | Unsuitable | 11,693.00 | 48,506 | 1.07 | 0.11 |
|  | Moderately suitable | 831.18 | 14,001 | 0.08 | 0.03 |
|  | Highly suitable | 1,032.92 | 86,055 | 0.09 | 0.19 |
| Pucheng Xian | Unsuitable | 25,710.08 | 107,621 | 2.36 | 0.24 |
|  | Moderately suitable | 2,307.94 | 34,043 | 0.21 | 0.08 |
|  | Highly suitable | 3,010.00 | 180,701 | 0.28 | 0.41 |
| Qingliu Xian | Unsuitable | 14,202.68 | 52,951 | 1.30 | 0.12 |
|  | Moderately suitable | 1,032.92 | 15,111 | 0.09 | 0.03 |
|  | Highly suitable | 1,355.71 | 76,378 | 0.12 | 0.17 |
| Quangang Qu | Unsuitable | 823.11 | 19,656 | 0.08 | 0.04 |
|  | Moderately suitable | 395.42 | 15,228 | 0.04 | 0.03 |
|  | Highly suitable | 1,202.39 | 284,046 | 0.11 | 0.64 |
| Quanzhou Economic and Technological Development Qu | Unsuitable | 0.00 | 0 | 0 | 0 |
|  | Moderately suitable | 0.00 | 0 | 0 | 0 |
|  | Highly suitable | 48.42 | 9,711 | 0 | 0.02 |
| Sanyuan Qu | Unsuitable | 6,068.42 | 19,085 | 0.56 | 0.04 |
|  | Moderately suitable | 314.72 | 5,832 | 0.03 | 0.01 |
|  | Highly suitable | 960.29 | 250,783 | 0.09 | 0.56 |
| Sha Xian | Unsuitable | 13,298.87 | 50,891 | 1.22 | 0.11 |
|  | Moderately suitable | 1,057.13 | 16,315 | 0.10 | 0.04 |
|  | Highly suitable | 2,001.29 | 152,238 | 0.18 | 0.34 |
| Shanghang Xian | Unsuitable | 18,285.95 | 7,3031 | 1.68 | 0.16 |
|  | Moderately suitable | 2,469.33 | 35,238 | 0.23 | 0.08 |
|  | Highly suitable | 4,890.24 | 292,239 | 0.45 | 0.66 |
| Shaowu Shi | Unsuitable | 22,675.87 | 59,506 | 2.08 | 0.13 |
|  | Moderately suitable | 1,024.85 | 13,406 | 0.09 | 0.03 |
|  | Highly suitable | 1,694.64 | 230,435 | 0.16 | 0.52 |
| Shishi Shi | Unsuitable | 112.98 | 49,347 | 0.01 | 0.11 |
|  | Moderately suitable | 8.07 | 333 | 0.00 | 0.00 |
|  | Highly suitable | 1,137.83 | 718,842 | 0.10 | 1.62 |
| Shouning Xian | Unsuitable | 10,506.75 | 59,256 | 0.96 | 0.13 |
|  | Moderately suitable | 1,420.27 | 25,948 | 0.13 | 0.06 |
|  | Highly suitable | 1,040.99 | 104,546 | 0.10 | 0.23 |
| Shunchang Xian | Unsuitable | 14,920.88 | 62,252 | 1.37 | 0.14 |
|  | Moderately suitable | 1,073.27 | 13,870 | 0.1 | 0.03 |
|  | Highly suitable | 1,928.66 | 133,199 | 0.18 | 0.3 |
| Siming Qu | Unsuitable | 96.84 | 175,976 | 0.01 | 0.4 |
|  | Moderately suitable | 24.21 | 889 | 0.00 | 0.00 |
|  | Highly suitable | 540.67 | 2,405,982 | 0.05 | 5.41 |
| Songxi Xian | Unsuitable | 7,553.24 | 28,133 | 0.69 | 0.06 |
|  | Moderately suitable | 726.27 | 10,719 | 0.07 | 0.02 |
|  | Highly suitable | 1,307.29 | 93,786 | 0.12 | 0.21 |
| Taijiang Qu | Unsuitable | 0.00 | 0 | 0 | 0 |
|  | Moderately suitable | 0.00 | 0 | 0 | 0 |
|  | Highly suitable | 250.16 | 669,914 | 0.02 | 1.51 |
| Taining Xian | Unsuitable | 12,338.58 | 46,215 | 1.13 | 0.10 |
|  | Moderately suitable | 613.30 | 8,261 | 0.06 | 0.02 |
|  | Highly suitable | 1,057.13 | 58,205 | 0.10 | 0.13 |
| Tongan Qu | Unsuitable | 3,332.79 | 40,961 | 0.31 | 0.09 |
|  | Moderately suitable | 564.88 | 31,738 | 0.05 | 0.07 |
|  | Highly suitable | 1,847.96 | 921,415 | 0.17 | 2.07 |
| Wuping Xian | Unsuitable | 18,778.20 | 79,164 | 1.72 | 0.18 |
|  | Moderately suitable | 1,791.47 | 25,529 | 0.16 | 0.06 |
|  | Highly suitable | 2,792.12 | 192,188 | 0.26 | 0.43 |
| Wiyishan Shi | Unsuitable | 22,764.63 | 60,792 | 2.09 | 0.14 |
|  | Moderately suitable | 952.23 | 14,475 | 0.09 | 0.03 |
|  | Highly suitable | 1,864.10 | 163,956 | 0.17 | 0.37 |
| Xiapu Xian | Unsuitable | 9,619.09 | 127,270 | 0.88 | 0.29 |
|  | Moderately suitable | 2,122.33 | 61,957 | 0.19 | 0.14 |
|  | Highly suitable | 1,404.13 | 230,215 | 0.13 | 0.52 |
| Xianyou Xian | Unsuitable | 10,216.24 | 87,613 | 0.94 | 0.20 |
|  | Moderately suitable | 2,227.24 | 81,591 | 0.20 | 0.18 |
|  | Highly suitable | 3,631.37 | 717,051 | 0.33 | 1.61 |
| Xiangcheng Qu | Unsuitable | 742.41 | 5,003 | 0.07 | 0.01 |
|  | Moderately suitable | 185.60 | 4,262 | 0.02 | 0.01 |
|  | Highly suitable | 1,363.78 | 603,306 | 0.12 | 1.36 |
| Xiang'an Qu | Unsuitable | 944.16 | 28,975 | 0.09 | 0.07 |
|  | Moderately suitable | 217.88 | 8,566 | 0.02 | 0.02 |
|  | Highly suitable | 1,807.617 | 520,491 | 0.17 | 1.17 |
| Xinluo Qu | Unsuitable | 19,690.08 | 63,792 | 1.80 | 0.14 |
|  | Moderately suitable | 1,484.83 | 42,475 | 0.14 | 0.1 |
|  | Highly suitable | 2,993.86 | 625,239 | 0.27 | 1.41 |
| Xiuyu Qu | Unsuitable | 774.69 | 81,244 | 0.07 | 0.18 |
|  | Moderately suitable | 72.63 | 2,101 | 0.01 | 0.00 |
|  | Highly suitable | 2,646.86 | 413,477 | 0.24 | 0.93 |
| Yanping Qu | Unsuitable | 19,181.69 | 86,968 | 1.76 | 0.20 |
|  | Moderately suitable | 1,670.43 | 26,190 | 0.15 | 0.06 |
|  | Highly suitable | 3,155.25 | 383,426 | 0.29 | 0.86 |
| Yong'an Shi | Unsuitable | 22,425.71 | 61,499 | 2.05 | 0.14 |
|  | Moderately suitable | 1,170.11 | 22,810 | 0.11 | 0.05 |
|  | Highly suitable | 2,888.95 | 314,341 | 0.26 | 0.71 |
| Yongchun Xian | Unsuitable | 8,110.05 | 73,120 | 0.74 | 0.16 |
|  | Moderately suitable | 3,001.93 | 103,241 | 0.28 | 0.23 |
|  | Highly suitable | 2,090.05 | 429,745 | 0.19 | 0.97 |
| Yongding Xian | Unsuitable | 14,477.05 | 58,043 | 1.33 | 0.13 |
|  | Moderately suitable | 1,387.99 | 24,260 | 0.13 | 0.05 |
|  | Highly suitable | 3,929.95 | 306,237 | 0.36 | 0.69 |
| Yongtai Xian | Unsuitable | 16,744.63 | 93,438 | 1.53 | 0.21 |
|  | Moderately suitable | 2,001.29 | 49,075 | 0.18 | 0.11 |
|  | Highly suitable | 1,557.45 | 180,469 | 0.14 | 0.41 |
| Youxi Xian | Unsuitable | 26,113.56 | 123,763 | 2.39 | 0.28 |
|  | Moderately suitable | 1,904.45 | 35,303 | 0.17 | 0.08 |
|  | Highly suitable | 2,525.82 | 212,366 | 0.23 | 0.48 |
| Yunxiao Xian | Unsuitable | 4,309.22 | 59,029 | 0.39 | 0.13 |
|  | Moderately suitable | 2,267.59 | 65,249 | 0.21 | 0.15 |
|  | Highly suitable | 2,469.33 | 355,394 | 0.23 | 0.80 |
| Zhangping Shi | Unsuitable | 23,854.04 | 84,140 | 2.19 | 0.19 |
|  | Moderately suitable | 976.43 | 18,729 | 0.09 | 0.04 |
|  | Highly suitable | 1,662.36 | 142,034 | 0.15 | 0.32 |
| Zhangpu Xian | Unsuitable | 8,432.84 | 130,735 | 0.77 | 0.29 |
|  | Moderately suitable | 3,001.93 | 82,901 | 0.28 | 0.19 |
|  | Highly suitable | 5,503.54 | 698,309 | 0.50 | 1.57 |
| Changle Shi | Unsuitable | 1,533.24 | 29,029 | 0.14 | 0.07 |
|  | Moderately suitable | 1,073.27 | 42,093 | 0.10 | 0.09 |
|  | Highly suitable | 3,324.72 | 792,783 | 0.30 | 1.78 |
| Changtai Xian | Unsuitable | 5,350.21 | 35,736 | 0.49 | 0.08 |
|  | Moderately suitable | 992.57 | 29,834 | 0.09 | 0.07 |
|  | Highly suitable | 1,509.03 | 154,760 | 0.14 | 0.35 |
| Changting Xian | Unsuitable | 21,860.83 | 81,715 | 2.00 | 0.18 |
|  | Moderately suitable | 2,041.63 | 29,287 | 0.19 | 0.07 |
|  | Highly suitable | 3,986.43 | 312,962 | 0.37 | 0.7 |
| Zhao'an Xian | Unsuitable | 4,769.19 | 71,365 | 0.44 | 0.16 |
|  | Moderately suitable | 2,194.96 | 68,391 | 0.20 | 0.15 |
|  | Highly suitable | 4,099.41 | 534,625 | 0.38 | 1.20 |
| Zherong Xian | Unsuitable | 4,034.85 | 26,964 | 0.37 | 0.06 |
|  | Moderately suitable | 556.81 | 11,665 | 0.05 | 0.03 |
|  | Highly suitable | 419.62 | 61,686 | 0.04 | 0.14 |
| Zhenghe Xian | Unsuitable | 13,540.96 | 59,018 | 1.24 | 0.13 |
|  | Moderately suitable | 1,016.78 | 19,007 | 0.09 | 0.04 |
|  | Highly suitable | 1,371.85 | 109,119 | 0.13 | 0.25 |
| Zhouning Xian | Unsuitable | 8,319.86 | 36,913 | 0.76 | 0.08 |
|  | Moderately suitable | 661.72 | 17,119 | 0.06 | 0.04 |
|  | Highly suitable | 556.81 | 69,941 | 0.05 | 0.16 |
